# Trichostatin A downregulates bromodomain and extra-terminal proteins to suppress osimertinib resistant non-small cell lung carcinoma

**DOI:** 10.1186/s12935-021-01914-y

**Published:** 2021-04-15

**Authors:** Yuting Meng, Xixi Qian, Li Zhao, Nan Li, Shengjie Wu, Baoan Chen, Tong Sun, Xuerong Wang

**Affiliations:** 1 Department of Pharmacology, Nanjing Medical University, 140 Hanzhong Road, Nanjing, 210029 Jiangsu Province China; 2 Department of Pharmacology, Sir Run Run Shaw Hospital, School of Medicine, Zhejiang University, Hangzhou, 310000 Zhejiang China; 3 Department of Hematology and Oncology, Zhongda Hospital, School of Medicine, Southeast University, Nanjing, 210009 Jiangsu China; 4 Laboratory of Human Functional Genomics of Jiangsu Province, Nanjing Medical University, 101 Longmiandadao, Nanjing, 211166 Jiangsu Province China

**Keywords:** Lung cancer, EGFR, Bromodomain and extra-terminal protein, Histone deacetylase

## Abstract

**Background:**

The third-generation epithelial growth factor receptor tyrosine kinase inhibitors (EGFR-TKIs) have shown significant therapeutic effects on patients with non-small cell lung carcinoma (NSCLC) who carry active EGFR mutations, as well as those who have developed acquired resistance to the first-generation of EGFR-TKIs due to the T790M mutation. However, most patients develop drug resistance after 8–10 months of treatment. Currently, the mechanism has not been well clarified, and new therapeutic strategies are urgently needed.

**Methods:**

Osimertinib resistant cell lines were established by culturing sensitive cells in chronically increasing doses of osimertinib. The anticancer effect of reagents was examined both in vitro and in vivo using the sulforhodamine B assay and a xenograft mouse model. The molecular signals were detected by western blotting. The combination effect was analyzed using CompuSyn software.

**Results:**

We found that bromodomain and extra-terminal proteins (BETs) were upregulated in osimertinib resistant (H1975-OR) cells compared with those in the paired parental cells (H1975-P), and that knockdown of BETs significantly inhibited the growth of H1975-OR cells. The BET inhibitor JQ1 also exhibited stronger growth-inhibitory effects on H1975-OR cells and a greater expression of BETs and the downstream effector c-Myc than were observed in H1975-P cells. The histone deacetylase (HDAC) inhibitor trichostatin A (TSA) showed stronger growth suppression in H1975-OR cells than in H1975-P cells, but vorinostat, another HDAC inhibitor, showed equal inhibitory efficacy in both cell types. Consistently, downregulation of BET and c-Myc expression was greater with TSA than with vorinostat. TSA restrained the growth of H1975-OR and H1975-P xenograft tumors. The combination of TSA and JQ1 showed synergistic growth-inhibitory effects in parallel with decreased BET and c-Myc expression in both H1975-OR and H1975-P cells and in xenograft nude mouse models. BETs were not upregulated in osimertinib resistant HCC827 cells compared with parental cells, while TSA and vorinostat exhibited equal inhibitory effects on both cell types.

**Conclusion:**

Upregulation of BETs contributed to the osimertinib resistance of H1975 cells. TSA downregulated BET expression and enhanced the growth inhibitory effect of JQ1 both in vitro and in vivo. Our findings provided new strategies for the treatment of osimertinib resistance.

## Background

Lung cancer is the leading cause of cancer related deaths worldwide [1]. A clinically significant subpopulation of patients with non-small cell lung cancer (NSCLC), especially women, non-smokers, and East Asians, have epidermal growth factor receptor (EGFR) mutations [2, 3]. EGFR tyrosine kinase inhibitors (EFGR-TKIs) have achieved clinically significant therapeutic effects, but patients can develop resistance after 8–10 months of TKI treatment [4]. The most common mechanism of resistance to the first-generation EGFR-TKIs is the gatekeeper mutation T790M, occurring in 60% of patients [5]. Third-generation EGFR-TKIs, such as osimertinib, rociletinib, and WZ4002, have shown their efficacy in counteracting the growth of EGFR T790M mutant tumors [6, 7]. Recently, osimertinib has been recommended as the first-line treatment for patients with advanced or metastatic NSCLC who carry EGFR-sensitive mutations or acquired T790M resistant mutations after using first or second-generation EGFR-TKIs [8]. However, an effective strategy is still needed for treatment of patients who have developed resistance to the third-generation EGFR-TKIs.

The widespread clinical use of osimertinib in patients with NSCLC has made acquired resistance an urgent issue. A range of studies have revealed some mechanisms of resistance, including EGFR C797S mutations, MET amplification, and sustained activation of the MAPK kinase pathway [9–11]. Clarifying the mechanism of osimertinib resistance would provide new therapeutic strategies for patients and facilitate the design of drugs that targeting key molecules involved in resistance.

One class of attractive targets are the bromodomain and extra-terminal proteins (BETs), which act as epigenetic “readers” that recognize and bind acetylated histones or other molecules to regulate gene expression transcriptionally [12]. BET proteins have been reported to promote tumor growth, metastasis, and drug resistance in a variety of cancer types, including colon, breast, and lung cancers [13, 14]. The inhibitors of BETs, and especially BRD4 inhibitors (JQ1 and I-BET151), have shown significant therapeutic efficacy in clinical trials in patients with malignant hematological cancers, such as multiple myeloma and leukemia [15–17]. However, the efficacy of BET inhibitors in lung cancer was not sufficient.

In a previous study, we reported that JQ1 targeting of BRD4 inhibited the growth of NSCLC via downregulation of eIF4E expression [18]. A recent report indicated that BET degraders suppressed the growth of NSCLC by upregulating Mcl-1 degradation and promoting apoptosis, but JQ1 did not show the same efficacy. Currently, the contribution of BETs to the resistance of osimertinib and the possibility of targeting BETs to restrain the growth of osimertinib resistant NSCLC remain to be established.

The functioning of BETs relies on histone acetylation as a key step [19]. Histone acetylation and deacetylation are regulated by histone deacetylase (HDAC) and histone acetyltransferase (HAT) enzymes, respectively [20]. Acetylation of histones enhances the transcription of tumor-promoting genes and facilitates cancer development [21]. Consequently, inhibitors of HDACs, such as trichostatin A (TSA), vorinostat, and MS-275, have been reported to suppress a variety of cancers, including lung, gastric, and breast cancers [22, 23]. Vorinostat has been proved effective against cutaneous T-cell lymphoma [24], but the effectiveness of HDAC inhibitors for suppression of osimertinib resistant NSCLC is unknown.

In this study, we examined BET expression in paired osimertinib sensitive and osimertinib resistant cell lines, evaluated the in vitro and in vivo inhibitory effects of BET inhibitors and an HDAC inhibitor on tumor growth, and explored the potential mechanisms. Our findings open up new avenues to potential therapeutic new targets and strategies for treatment of osimertinib resistant NSCLC.

## Methods

### Reagents

The following antibodies were purchased: anti-BRD4 (E2A7X) from Cell Signaling Technology, anti-BRD2 (D89B4) from Cell Signaling Technology, anti-BRD3 (2088C30) from Abcam Technology, anti-c-Myc (A19032) from ABclonal Technology, anti-c-Met (25869-1-AP) from Proteintech Technology, anti-EGFR (2232) from Cell Signaling Technology, and anti-GAPDH (AP0063) from Bioworld Technology Inc. All antibodies were utilized at 1:1000 dilutions, except GAPDH (1:10,000). JQ1 (HY-13030) and (2-Hydroxypropyl)-β-cyclodextrin (HY-101103) were purchased from Haoyuan Chemexpress Co., Ltd. Trichostatin A (T6270) was purchased from Target Mol, Inc. Vorinostat (T1583) was purchased from Target Mol, Inc. Osimertinib (HY-15772) was purchased from MedChemExpress (Monmouth Junction, NJ, USA). Reagents were dissolved in DMSO at 20 mmol/L, stored at -20℃, and diluted just before use. Lipofectamine 2000® transfection reagent (11668-019) was purchased from Life Technologies Co. Invitrogen (Carlsbad, CA, USA).

### Cell lines and culture conditions

The H1975 and HCC827 EGFR-mutant NSCLC cell lines were kindly provided by Dr. Shi-Yong Sun (Emory University, USA). The H1975-OR and HCC827-OR osimertinib resistant cell lines were newly established in our laboratory by exposing H1975 and HCC827 cells to gradually increasing concentrations of osimertinib (from 1 to 1000 nmol/L) for approximately 6 months. In detail, the cells were first exposed to 1 nmol/L osimertinib, then the drug was withdrawn for recovery when the survival rate decreased to 30%. The dose increase was 5 nmol/L at first and then changed to 20 nmol/L and 50 nmol/L when the IC50 increased by 50-fold and 500-fold, respectively. Untreated cells cultured in parallel were defined as parental cells (H1975-P and HCC827-P). The resistance index (RI) was calculated as the ratio of the IC50 values of resistant versus parental cells. Cells were cultured in RPMI1640 medium (Gibco) supplemented with 10% fetal bovine serum (Lonsera) at 37 °C in a humidified 5% CO_2_ atmosphere.

### Gene knockdown

All siRNAs were obtained from Shanghai GenePharm. Cells were transfected with siRNAs at a final concentration of 100 nmol/L using Lipofectamine2000 for subsequent experiments. The following siRNAs sequences were used: Control siRNA that target: 5′-UUCUCCGAACGUGUCACGUTT-3′; 5′-ACGUGACACGUUCGGAGAATT-3′; BRD4 siRNA that target:5′-CUCCCUGAUUACUAUAAGATT-3′; 5′-GCACAAUCAAGUCUAAACUTT-3′; 5′-GGAGAUGACAUAGUCUUAATT-3′, BRD3 siRNA that target: 5′-GUGCAAGCGAAUGUAUGCATT-3′; 5′-CGGAUGUUCUCGAAUUGCUTT-3′; 5′-GUAGUGCACAUCAUCCAAUTT-3′, BRD2 siRNA that target: 5′-CAGCUGCAAUACCUACACATT-3′; 5′-GACUUCUCAAGUCCUUGCATT-3′; 5′-GGACAGCUCAAUUCUACUATT-3′; c-Myc siRNA that target: 5′-GGTCAGAGTCTGGATCACC-3′; 5′-CGAGCTAAAACGGAGCTTT-3′; 5′-GCTTGTACCTGCAGGATCT-3’.

### Sulforhodamine B assay

Cells were seeded in 96-well plates at 2500 cells/well and treated on the second day with different drug concentrations for 3 days. Cell numbers were determined by the sulforhodamine B (SRB) staining, as described previously [25].

### Western blot analysis

Whole-cell protein lysates were prepared and subjected to western blotting as described previously [26]. The protein expression levels were quantified and are presented under the blot images. The index of density (IOD = density × area) for each blot was first obtained by ImageJ and then the expression level of each protein was calculated by the formula: IOD ratio = IOD _(Target protein)_/IOD _(GAPDH)_. The fold change for each treatment compared to the control was then calculated as: Fold change = IOD ratio _(Treatment)_/IOD ratio _(Control)_.

### Quantitative real-time polymerase chain reaction (qRT-PCR)

Total RNA from cells was extracted using TRIzol® reagent (1596–026) from Invitrogen Life Technologies (Carlsbad, CA, USA). Crushed, snap-frozen tumor tissues were homogenized in TRIzol. Cells were harvested for qRT-PCR as we described previously [27]. The following primer sequences were used for this study: BRD4, F: AGCAGCAACAGCAATGTGAG and R: GCTTGCACTTGTCCTCTTCC; BRD3, F: CGGAAGCTCCAGGACGTGTT and R: GGAGCCACCTTGGCCTTCTT; BRD2, F: CAGGAACAGCTTCGGGCAGT; R: TCATGGGCCTGCTCTCTTCC; GAPDH, F: ATGGGGAAGGTGAAGGTCG; R: GGGGTCATTGGCAACAACAATA; c-Myc, F: AAACACAAACTTGAACAGCTAC; R: ATTTGAGGCAGTTTACATTATGG.

### Lung cancer xenografts in nude mice

All experiments were conducted in accordance with protocols approved by the Nanjing Medical University at Animal Care and Use Committee. Female athymic (nu/nu) mice, 4 to 5 weeks of age, were purchased from the Model Animal Research Center of Nanjing University. The mice were kept on a 12 h light–dark cycle with free access to food and water. Preliminary experimental results showed that the growth rate of subcutaneous tumors was slower with H1975-OR cells than with H1975-P cells. H1975-OR cells ( 5 × 10^6^cells/mouse) were injected subcutaneously into the right flank regions of nude mice. Four weeks later, H1975-P cells (5 × 10^6^cells/mouse) were injected subcutaneously into the right flank regions of another batch of nude mice purchased simultaneously. TSA (0.5 mg/kg, daily) and the vehicle control were administered on the second day after inoculation by intraperitoneal (i.p.) injection (n = 7 mice/group). For the combination study, mice were divided into four groups and treated on the second day after cell injection (n = 7 mice/group) with vehicle (control: DMSO + 10% β-cyclodextrin), TSA 0.5 mg/kg, JQ1 100 mg/kg, or TSA + JQ1 by intraperitoneal (i.p.) injection. Tumor diameters and body weights were measured every three days. After 21 days, the mice were sacrificed and the tumors were removed and weighed. Tumor volume was calculated with the formula V = π (length × width^2^)/6.

### Statistical analysis

The data are presented as the mean ± SD from triplicate or quadruplicate samples. The results are representative of at least three independent experiments. Statistical significance between two groups was analyzed using two-tailed unpaired Student t tests. Multiple groups were compared using one-way ANOVA. GraphPad software was used for data analysis. The IC50 values were determined by GraphPad. The combination index (CI) was calculated using CompuSyn. The results were considered statistically significant at *p* < 0.05.

## Results

### Osimertinib resistant cells showed elevated expression of BETs.

**Fig. 1:**
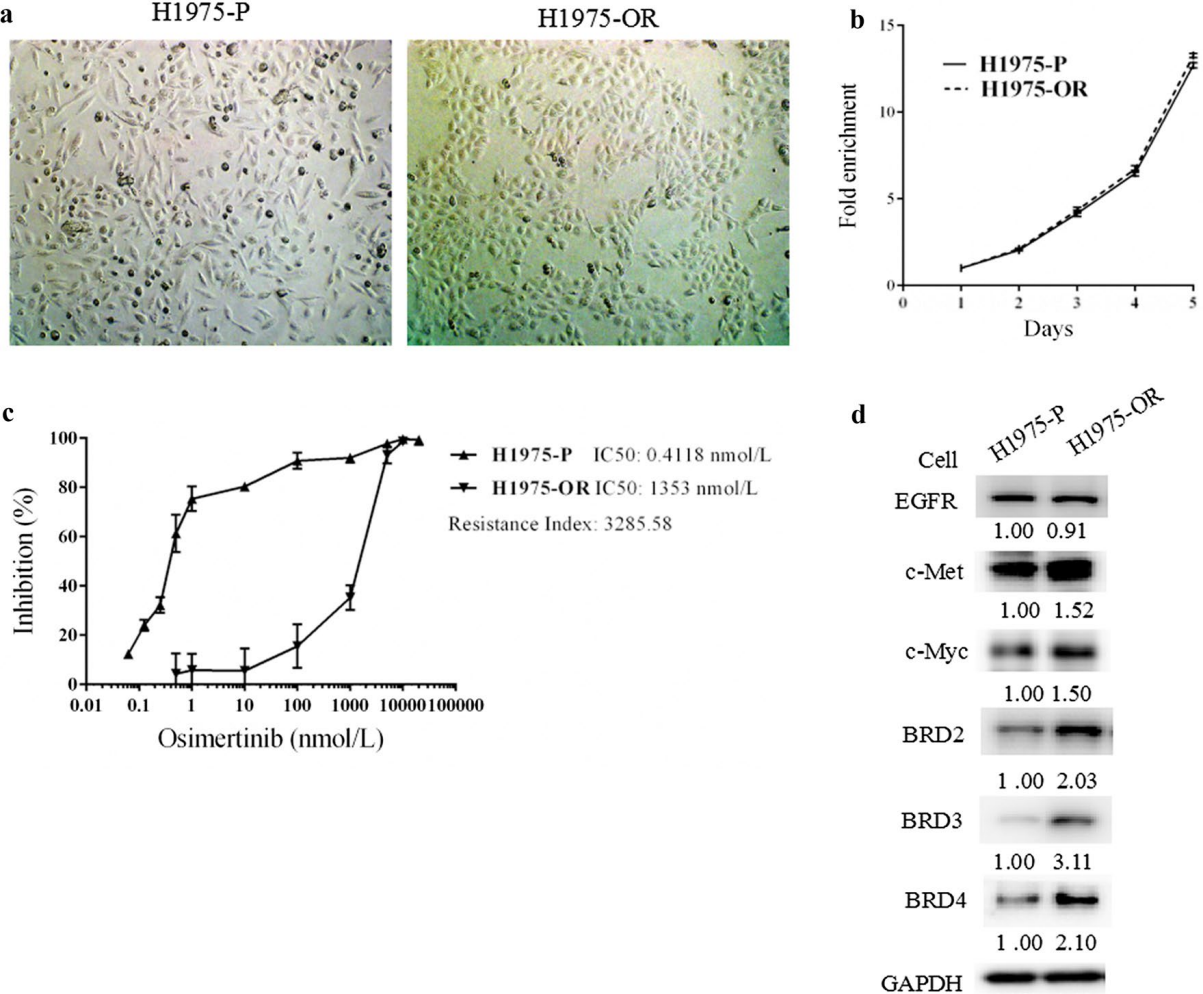
BETs were upregulated in osimertinib resistant (H1975-OR) cells compared to osimertinib sensitive parental (H1975-P) cells. A and B, Photos (**a**) and growth curve (**b**) of H1975-OR and H1975-P cells. Magnification, 100 × . **c**, **d** H1975-OR and H1975-P were treated with osimertinib for 3 days and subjected to sulforhodamine B (SRB) assays (**c**), or were cultured for 24 h and subjected to western blot analysis (**d**). Points, mean; bars, SD; *, *P* < 0.05

We explored the mechanism of osimertinib resistance by first establishing osimertinib resistant cell lines (H1975-osimertinib resistant, H1975-OR) using an osimertinib sensitive cell line H1975 (H1975-parental, H1975-P), which harbors EGFR L858R and T790M mutations resulting in constitutively activated EGFR signaling and shows sensitivity to osimertinib but resistance to the first-line EGFR-TKI erlotinib. H1975-OR cells were smaller in size than the H1975-P cells (Fig. 1a), but the growth rates were comparable between the two cell lines, as determined by SRB assays (Fig. 1b). The IC50 of osimertinib in H1975-OR cells dramatically increased to 1.35 μmol/L compared to 0.41 nmol/L in H1975-P cells with a resistance index (RI) of 3285.58 (Fig. 1c). Whole genome sequencing revealed an EGFR C797S mutation in 98.0% of the H1975-OR cells, but not in the H1975-P cells. The total EGFR protein levels were comparable in the parental and resistant cells (Fig. 1d). The C797S mutation results in insensitivity of EGFR to osimertinib but retention of EGFR activity; therefore, we suspected that EGFR signaling pathway was constitutively activated in the H975-OR cells.

Notably, we found that the protein levels of BETs, including BRD2, BRD3, and BRD4, were significantly higher in the H1975-OR cells than in the H1975-P cells (Fig. 1d). Other important downstream signaling molecules that are responsible for the resistance of first-line EGFR inhibitors, such as c-Myc and c-Met, were also increased significantly in the H1975-OR cells. These data indicated that the activation of BET signaling may contribute to osimertinib resistance.

### Knockdown of BETs inhibited the growth of H1975-OR cells.

**Fig. 2:**
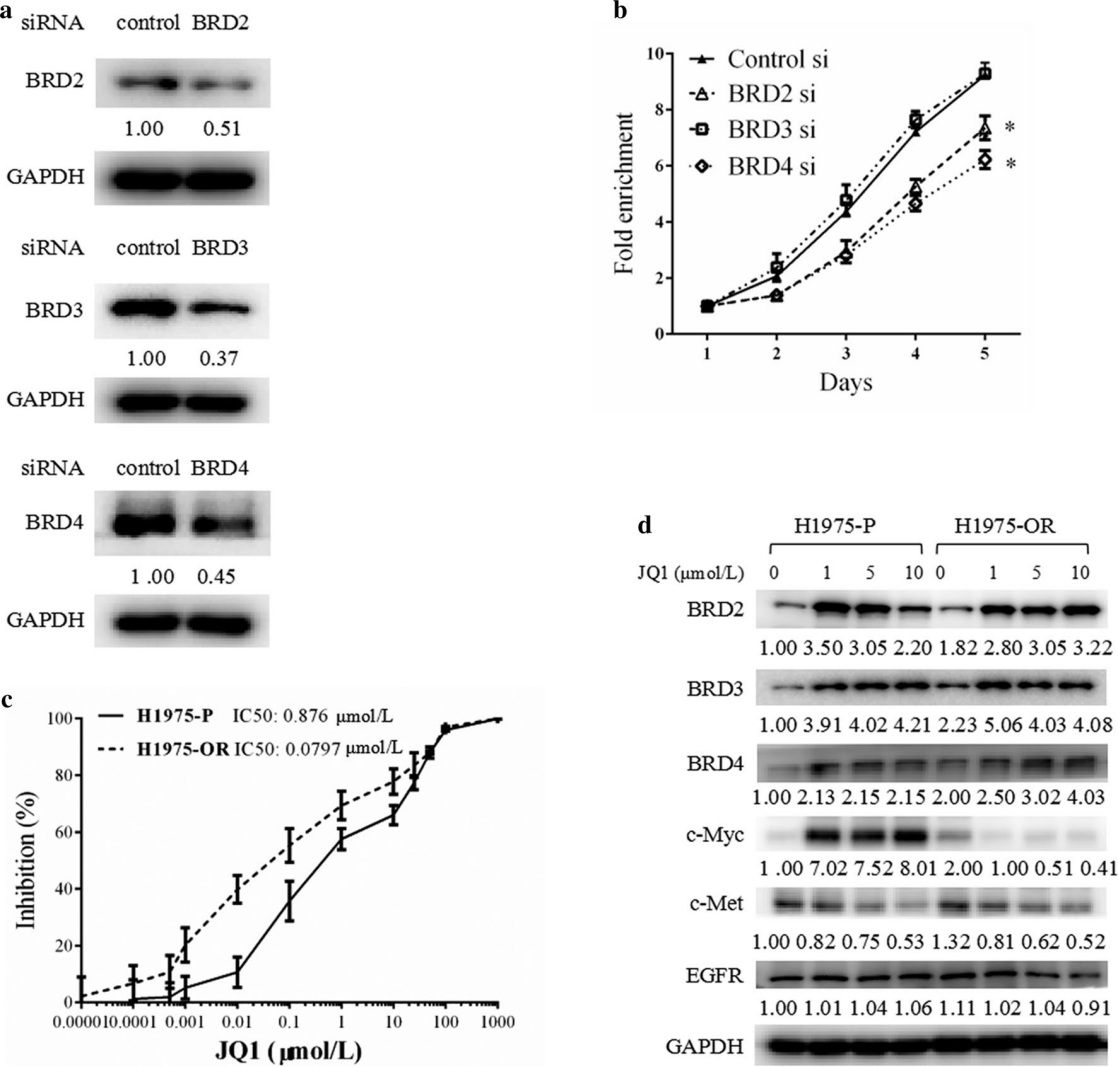
Knockdown of BET expression or inhibition of BET activity suppressed the growth of osimertinib resistant cells. **a**, **b** osimertinib resistant (H1975-OR) cells were transfected with siRNAs pools that specifically target BRD2, 3, or 4 for 24 h, followed by another 24 h incubation before being subjected to western blot analysis (**a**) or reseeded to 96-well plates for a 5-day SRB assay (**b**). **c**, **d** osimertinib sensitive parental (H1975-P) cells and H1975-OR cells were either treated with JQ1 for 3 days and subjected to sulforhodamine B (SRB) assays (**c**) or treated with JQ1 (1, 5, 10 μmol/L) for 24 h and subjected to western blot analysis (**d**). Points, mean; bars, SD; *, *P* < 0.05

We also explored the consequences of targeting BETs in H1975 cells. We knocked down BET expression using siRNAs targeting BRD2, BRD3, or BRD4 individually and examined the effect of BET depletion on cell growth. Western blot analysis confirmed the successful silencing of BET protein expression (Fig. 2a). The SRB assay results showed that knockdown of BRD2 and BRD4, but not of BRD3, significantly inhibited the growth of H1975-OR cells compared with cells treated with control siRNAs (Fig. 2b).

We also tested the effect of JQ1, a BET inhibitor that mainly targets BRD4. JQ1 treatment resulted in a greater growth inhibition in the H1975-OR cells than in the H1975-P cells, with IC50 values of 0.079 and 0.876 μmol/L, respectively (Fig. 2c). However, we noted that JQ1 increased the expression of all three BETs, which may ameliorate its anticancer efficacy (Fig. 2d). JQ1 treatment also markedly upregulated c-Myc expression in H1975-P cells but downregulated c-Myc expression in H1975-OR cells (Fig. 2d), whereas c-Met was downregulated in H1975-P cells and EGFR expression was unaltered in either cell type (Fig. 2d). These data suggest that targeting BETs using either siRNA or inhibitors could suppress the growth of osimertinib-resistance cells, but JQ1 may ameliorate its own anticancer efficacy by upregulating BETs under both osimertinib sensitive and osimertinib resistant cells and by upregulating c-Myc in osimertinib sensitive cells.

### The HDAC inhibitors TSA and vorinostat downregulated BET expression and inhibited the growth of osimertinib resistant cells

**Fig. 3:**
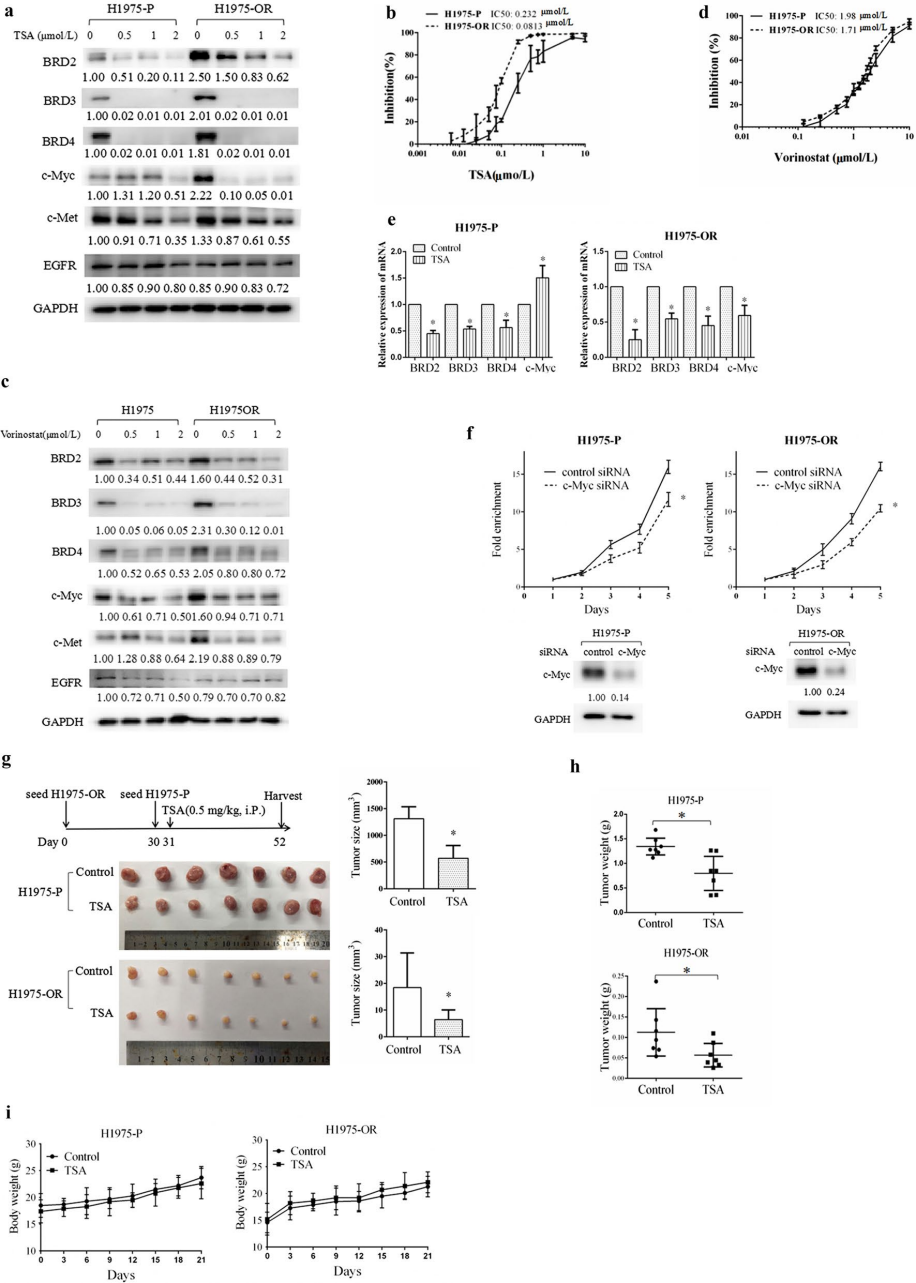
The histone deacetylase (HDAC) inhibitor trichostatin (TSA) downregulated BET expression and suppressed the growth of osimertinib resistant cells and xenograft tumors. **a**–**d** Osimertinib sensitive parental (H1975-P) and osimertinib resistant (H1975-OR) cells were either treated with TSA (**a**) or vorinostat (**b**) for 24 h and subjected to western blot analysis or treated with TSA (**c**) or vorinostat (**d**) for 3 days and subjected to sulforhodamine B (SRB) assays as indicated. E, The mRNA levels of BETs under TSA (1 μmol/L) treatment for 6 h. F, Cells were transfected with c-Myc or control siRNAs as indicated, and subjected to SRB assays and Western blotting. Columns, mean; Points, mean; bars, SD; *, *P* < 0.05. **g**–**i** A schematic diagram of experiment design, the tumor size (**g**), tumor weight (**h**), and body weight (**i**) are indicated. TSA (0.5 mg/kg/d) or vehicle was administered for 21 days. *G* columns, mean. *H* points, individual tumor weight; horizontal line, mean tumor weight. *I* points, mean. Bars, SD; *, *P* < 0.05

The BETs are DNA readers and can promote cell growth through epigenetic regulation. BETs activate gene promoters by binding with acetylated histones, thereby facilitating the transcription of oncogenes. However, their own transcriptional regulation still remains elusive. We found that treatment of either H1975-P or H1975-OR cells with TSA, a non-selective HDAC inhibitor that mainly works on Class I and II HDACs, significantly downregulated the expressions of BRD2, BRD3, and BRD4 (Fig. 3a). Moreover, the expression of c-Myc and c-Met, but not EGFR, decreased dramatically after TSA treatment (Fig. 3a), but the growth inhibitory action of TSA was stronger in H1975-OR than in H1975-P cells, with an IC50 values of 0.0813 and 0.232 μmol/L, respectively (Fig. 3b).

Treatment of the two cell types with vorinostat, a clinically used HDAC inhibitor that specifically targets HDAC1, 2, 3 and 6, downregulated BET expression and the downstream c-Myc and c-Met expression levels, but to a lesser extent than that observed with TSA (Fig. 3c). The H1975-OR and H1975-P cells exhibited equal sensitivity to vorinostat, suggesting the importance of BET signaling in osimertinib resistance (Fig. 3d).

We further examined the BET mRNA levels following TSA treatment and observed that BET levels significantly decreased after TSA treated for only 6 h, suggesting that BETs was downregulated directly in transcriptional or post-transcriptional levels (Fig. 3e). Since c-Myc was drastically downregulated by TSA treatment in resistant cells, we examined the effect of c-Myc in osimertinib resistance. We found that c-Myc silencing using siRNAs significantly suppressed the growth of both H1975-OR and H1975-P cells, suggesting the important role of c-Myc in osimertinib resistance (Fig. 3f).

We then evaluated the effect of TSA in a xenograft nude mouse model. TSA treatment significantly suppressed the growth of both the H1975-OR and the H1975-P tumors. The average size and weight of the H1975-P tumors were significantly smaller with TSA treatment than without TSA treatment (*p* < 0.05) (Fig. 3g, h). The body weights of the mice were comparable between these two groups, indicating the dose of TSA were tolerable (Fig. 3i). The size and weight of the H1975-P and H1975-OR tumors were not compared because the H1975-OR tumors grew more slowly and were smaller than the H1975-P tumors when the experiment ended.

These findings suggest that HDAC inhibitors downregulated the expression of BETs and the downstream oncogenic signals to suppress the growth of osimertinib resistant cells both in vitro and in vivo.

### Combination of HDACs inhibitors with JQ1 exhibited synergistic growth-inhibitory effect on osimertinib sensitive and resistant cells and xenograft tumors

**Fig. 4:**
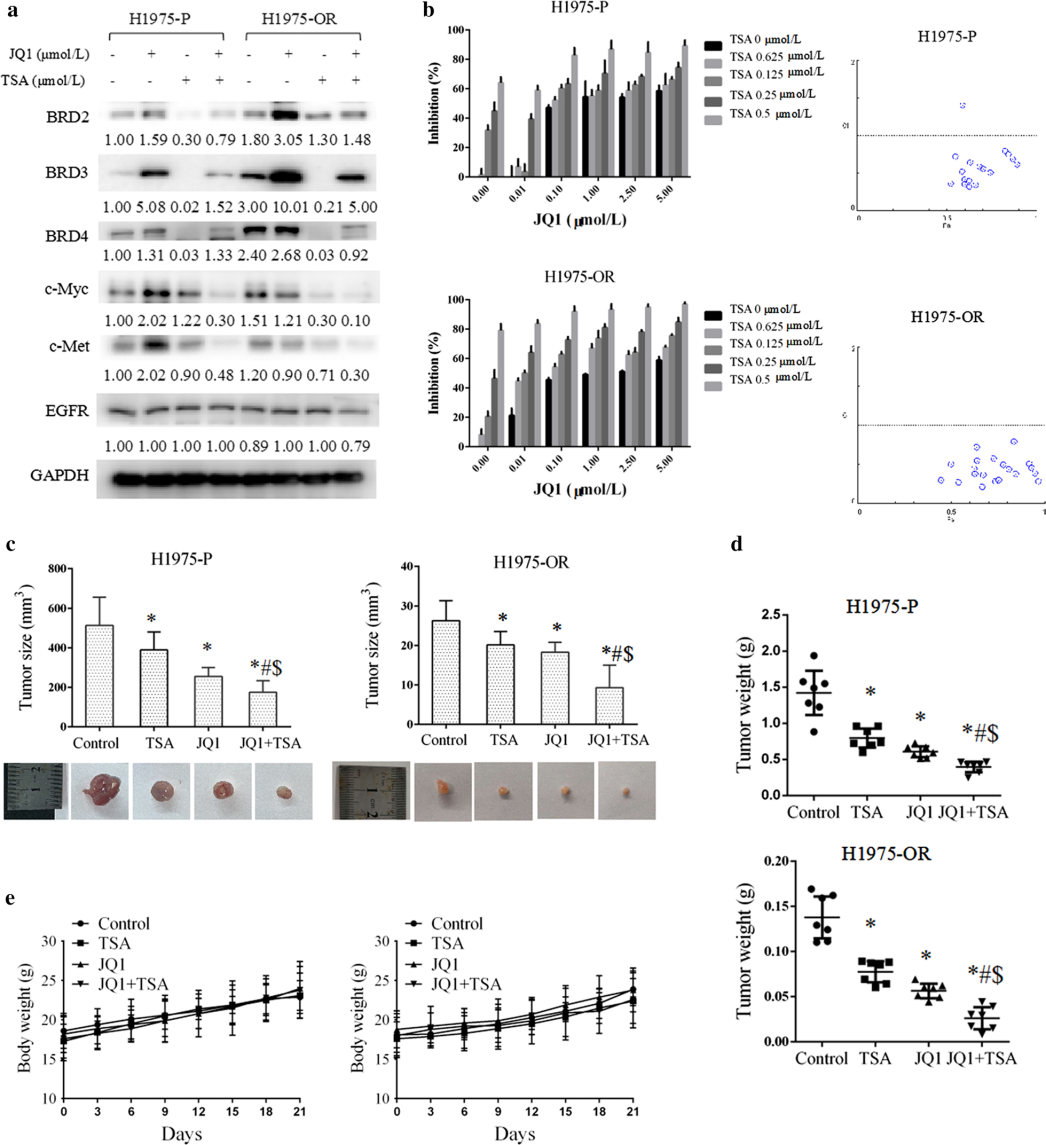
The combination of trichostatin A (TSA) and JQ1 synergistically inhibited the growth of osimertinib sensitive and resistant H1975 cells. **a** Osimertinib resistant (H1975-OR) and osimertinib sensitive parental (H1975-P) cells were treated with TSA (2 μmol/L), JQ1 (10 μmol/L), or their combination for 24 h, and then subjected to western blot analysis. **b** Cells were treated with TSA, JQ1, or their combination in different concentrations as indicated for 3 days and then subjected to sulforhodamine B (SRB) assays, followed by combination index assays using CompuSyn software. Points, mean; bars, SD. **c**–**e**, The tumor size (**c**), tumor weight (**d**), and body weight (**e**) of the xenograft mouse model are presented. The vehicle control, TSA (0.5 mg/kg/d), JQ1 (100 mg/kg/d), or their combination was administered for 21 day. In C: columns, mean. In D: points, individual tumor weight; horizontal line, mean tumor weight. In *E* Points, mean; bars, SD; *, *P* < 0.05 vs. control; #, *P* < 0.05 vs. TSA; $, *P* < 0.05 vs. JQ1

TSA downregulated BET and c-Myc expressions in both H1975-P and H1975-OR cells; therefore, we examined the effect of a combination of TSA and JQ1. As shown in Fig. 4a, TSA significantly decreased the induction of BRD2, BRD3, and BRD4 protein levels by JQ1 in both H1975-P and H1975-OR cells. The combination treatment of TSA and JQ1 further downregulated c-Myc and c-Met protein levels compared with each individual single-agent treatment (Fig. 4a). The combined use of TSA with JQ1 also synergistically suppressed cell growth as determined by the combination index (CI < 1) (Fig. 4b).

We then examined their combination on the growth of H1975-P and H1975-OR tumors in the xenograft mouse model. We observed that JQ1 (100 mg/kg, i.p daily) or TSA (0.5 mg/kg, i.p daily) significantly decreased tumor size and tumor weight compared with the vehicle control. The combination of JQ1 and TSA resulted in a synergistic restraint of tumor growth beyond that achieved by either treatment alone with statistical significance (*P* < 0.05) (Fig. 4c, d). No obvious toxicity was observed, based on the comparable body weights of the mice in the different groups (Fig. 4e). These data suggest that the combined use of TSA and JQ1 has a better therapeutic effect on osimertinib resistance and that the mechanism at least partially involves the suppression of BET-mediated signaling.

### HDAC inhibitors downregulated BET expressions and suppressed the growth of osimertinib sensitive and resistant HCC827 cells

**Fig. 5:**
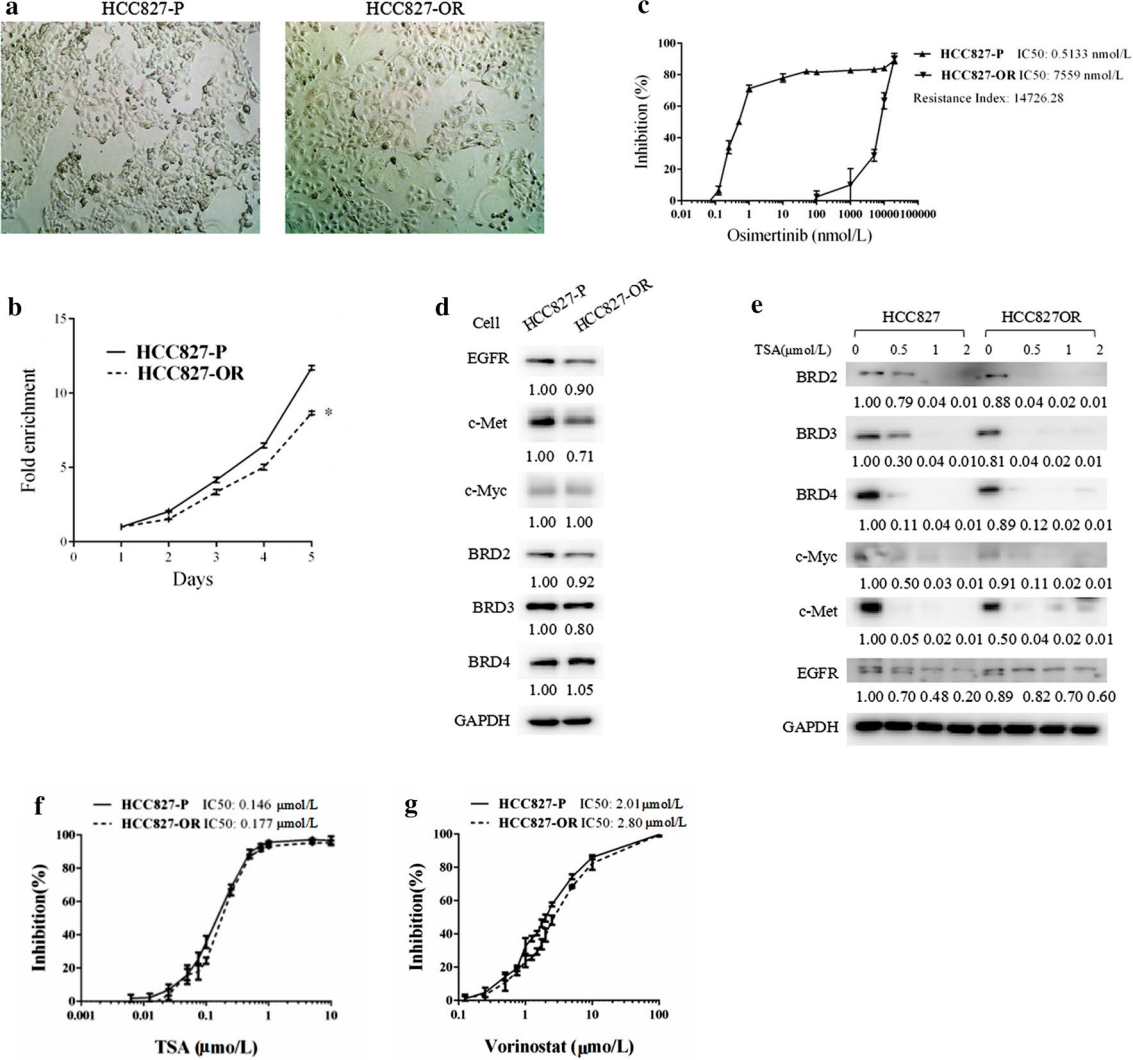
HDAC inhibitor treatment suppressed the growth of osimertinib resistant (HCC827-OR) and osimertinib sensitive parental (HCC827-P) cells. **a**, **b** Photos (**a**) and growth curve (**b**) of HCC827-OR and HCC827-P cells. Magnification, 100×. **c**, **d** Cells were treated with osimertinib for 3 days and subjected to sulforhodamine B (SRB) assays (**c**), or were cultured for 24 h and subjected to western blot analysis (**d**). E, HCC827-OR and HCC827-P cells were treated with TSA (0.5, 1, 2 μmol/L) for 24 h, and subjected to western blot analysis. **f**, **g**, HCC827 -OR and H1975-P cells were treated with TSA (**f**) or vorinostat (**g**) for 3 days and subjected to SRB assay. Points, mean; bars, SD

We also tested the effect of HDAC inhibitors on another pair of osimertinib sensitive (HCC827-P) and resistant (HCC827-OR) cells. The HCC827 cells harboring the deletion of exon 19 (Del 19), but no T790M mutation, were sensitive to both first-generation EGFR-TKIs, such as erlotinib and gefitinib, and the third-generation EGFR-TKI osimertinib. This cell type could therefore mimic those patients who carry a sensitive EGFR-TKI mutation or who develop resistance to first-line EGFR-TKIs but lack the T790M mutation. The HCC827-OR and HCC827-P cells were identical in phenotype, but the growth was slower for HCC827-OR cells than for HCC827-P cells (Fig. 5a, b). Whole genome sequencing did not reveal the EGFR C797S mutation in either of the two cell lines. The RI of HCC827-OR for osimertinib was 14,726.28 (IC50 of 0.5133 vs. 7559 nmol/L), indicating that the HCC827-OR cells were resistant to osimertinib (Fig. 5c). However, we did not observe any upregulation of BETs in the HCC827-OR cells above the expression in the HCC827-P cells (Fig. 5d).

The expressions of c-Myc, c-Met, and EGFR were also not increased (Fig. 5d). TSA dramatically downregulated BET expression, as well as that of c-Myc, c-Met, and EGFR in both HCC827-P and HCC827-OR cells (Fig. 5e). The HCC827-OR cells exhibited equal sensitivity to TSA and vorinostat (Fig. 5f, g). These findings suggest that HDAC inhibitors likely inhibit the growth of sensitive and resistant HCC827 cells by a different mechanism that that functioning in H1975 cells.

## Discussion

Osimertinib is a third-generation EGFR-TKI that is highly selective for EGFR-activating mutations, as well as for the EGFR T790M mutation, in patients with advanced NSCLC [28]. Despite the documented efficacy of osimertinib in first- and second-line settings, patients inevitably develop resistance [29]. In this study, we found that TSA can inhibit tumor growth both in vivo and in vitro. More importantly, TSA is more effective against H1975 osimertinib resistant cells than against the parental osimertinib sensitive cells, although TSA exhibited the same growth inhibitory effect on both the HCC827 resistant and parental cells. HDACs are well recognized to play crucial roles in cancer by deacetylating various histone and nonhistone proteins involved in the regulation of the cell cycle, apoptosis, DNA-damage responses, metastasis, angiogenesis, autophagy, and other cellular processes [30–32]. Another notable point is that the HCC827 cells did not contain C797S and T790M mutations; therefore, they may represent those patients with EGFR active mutations (Exon 19 delete) that are suitable for both first-line and third-line EGFR-TKIs but are administered osimertinib as the priority therapy. These findings indicate a potentially stronger inhibitory efficacy of TSA in osimertinib resistant patients with both T790M and C797S mutations of EGFR, indicating new therapeutic strategies.

We propose that acetylation of some proteins specifically enhanced by the EGFR signaling pathways (such as the C797S active mutation) may contribute to osimertinib resistance. However, unfortunately, the clinically used reagent vorinostat showed equal efficacy to osimertinib in both sensitive and resistant cells. The different effects of TSA on these two different H1975 and HCC827 drug-resistant cell lines deserve further study. For example, RNA sequencing analysis of EGFR-TKI-treated HCC827 cells may clarify the mechanism of osimertinib resistance.

Interestingly, JQ1 exhibited a stronger growth-inhibitory effect on H1975 osimertinib resistant cells than on osimertinib sensitive cells. We suspect that this may reflect the different BET basal protein levels in the two cell types. Previous reports have shown that the levels of BET proteins are higher in H1975 cells than in a panel of NSCLC cell lines, including H157, H1299, H1650, H460, H1972, and PC-9 cells [33]. In this study, we showed that H1975-OR cells have even higher BET protein levels than are found in H1975-P cells, suggesting that H1975-OR cells may be more sensitive to JQ1. Zong et al. [33] reported that BET protein levels were correlated positively with high sensitivity to BET degraders (a novel class of drugs that work by inducing BET protein degradation), such as ZBC260 and dBET, but not with JQ1. Our finding that JQ1 induced an upregulation of BET protein levels may partially explain the unfavorable anticancer activity of JQ1 in lung cancer. We also revealed that TSA drastically decreased both basal BET protein levels and the BET upregulation induced by JQ1; therefore, TSA exhibited significant growth inhibitory effects when used alone and it synergized the JQ1 effects when used in combination. Currently, we are uncertain whether the higher BET protein levels observed in H1975-OR cells determine their higher sensitivity compared to H1975-P cells; however, the potential mechanism deserves further study and may provide more efficient therapeutic strategies for overcoming osimertinib resistance.

Some studies have reported a transcriptional downregulation of *c-Myc* by JQ1 treatment by direct targeting of BRD4 in certain types of cancer, including lung cancer [34]. In this study, we observed that JQ1 inhibited the activity of BETs, but simultaneously upregulated BET expression, resulting in an upregulation of c-Myc in H1975 parental cells. However, in H1975 resistant cells, JQ1 significantly downregulated c-Myc levels, in parallel with increased BET protein levels, suggesting that c-Myc alteration may also be caused by some factors other than BETs. Another possibility is that elevation of the basal BET levels in H1975 resistant cells prevented further c-Myc upregulation of BET levels in response to JQ1 treatment and instead resulted in an indirect consequence of downregulation. However, the use of TSA, which almost completely eliminated BET proteins, led to downregulation of a portion of c-Myc due to the decreased BET protein levels. c-Myc silencing suppressed the growth of H1975-OR cells. Our findings indicate that c-Myc may be a key downstream effector of BETs that contributes to osimertinib resistance.

Previously, c-Met was reported to contribute to the resistance of the first-line EGFR-TKI and to be downregulated by BRD4 inhibitors [35]. Our results show that c-Met expression was higher in osimertinib resistant cells than in the parental cells. However, JQ1 or TSA reduced the c-Met levels in resistant H1975 cells to a lesser extent than in parental cells. This finding is inconsistent with the results showing that resistant cells were more sensitive to JQ1 or TSA treatments and indicates that c-Met is not a key factor in osimertinib resistance.

One point to note is that the HDAC inhibitor, TSA, almost completely eliminated BET expression in both osimertinib resistant and parental cells, whereas vorinostat only partially decreased the BET levels. The finding that osimertinib sensitive and osimertinib resistant cells exhibited equal sensitivity to vorinostat is somewhat disappointing, since vorinostat is a clinically approved medicine. However, these results suggest that the combination of BET inhibitors and HDAC inhibitors may benefit patients with osimertinib resistance.

## Conclusion

In summary, the upregulation of BETs in osimertinib resistant cells may contribute to resistance to this drug. TSA and JQ1 showed strong growth-inhibitory effects on osimertinib resistant NSCLCs via downregulation of BET expression and BET activity, respectively. The combination of JQ1 and TSA showed synergistic inhibitory efficacy. These findings partially clarified the mechanism of osimertinib resistance and provide potential new strategies for NSCLC therapy.

## Data Availability

Not applicable.

## Abbreviations

EGFR-TKIs: Epithelial growth factor receptor tyrosine kinase inhibitors;
NSCLC: Non-small cell lung carcinoma;
BETs: Bromodomain and extra-terminal proteins;
HDAC: Histone deacetylase;
TSA: Trichostain A;
HAT: Histone acetyltransferase
